# Effects of the Openness to Experience Polygenic Score on Cortical Thickness and Functional Connectivity

**DOI:** 10.3389/fnins.2020.607912

**Published:** 2021-01-11

**Authors:** Zhiting Ren, Cheng Liu, Jie Meng, Qiang Liu, Liang Shi, Xinran Wu, Li Song, Jiang Qiu

**Affiliations:** ^1^Key Laboratory of Cognition and Personality (SWU), Ministry of Education, Chongqing, China; ^2^Faculty of Psychology, Southwest University (SWU), Chongqing, China

**Keywords:** openness to experience, genome-wide polygenic scores, cortical thickness, functional connectivity, brain structure

## Abstract

Openness to experience (OTE) has relatively stable and heritable characteristics. Previous studies have used candidate gene approaches to explore the genetic mechanisms of OTE, but genome-wide polygenic scores have a greater genetic effect than other genetic analysis methods, and previous studies have never examined the potential effect of OTE on this cumulative effect at the level of the brain mechanism. In the present study, we aim to explore the associations between polygenic scores (PGSs) of OTE and brain structure and functions. First, the results of PGSs of OTE at seven different thresholds were calculated in a large Chinese sample (*N* = 586). Then, we determined the associations between PGSs of OTE and cortical thickness and functional connectivity. The results showed that PGSs of OTE was negatively correlated with the thickness of the fusiform gyrus, and PGSs of OTE were negatively associated with the functional connectivity between the left intraparietal sulcus (IPS) and the right posterior occipital lobe. These findings may suggest that the brain structure of fusiform gyrus and brain functions of IPS and posterior occipital lobe are partly regulated by OTE-related genetic factors.

## Introduction

Openness to experience (OTE) is a dimension derived from personality that reflects individual differences in cognitive exploration of reasoning and perception (DeYoung, 2014). Previous studies have usually regarded OTE as an element of the Big Five Personality Model (McCrae, 1993). Individuals with high OTE generally show a greater curiosity for new things, a desire to explore, a sense of satisfaction derived from new knowledge and experiences, and a high tolerance for strange situations (McCrae and Costa, 1985). OTE is primarily related to individual intelligence and cognitive style, such as imagination and creative cognition (DeYoung et al., 2008; DeYoung and Allen, 2019). Artists and intellectuals scored higher on OTE in previous study (DeYoung, 2014). Some studies demonstrated that differences in OTE and intelligence significantly predict creative achievements in art and science (Kaufman et al., 2016). Moreover, OTE has been showed to be associated with positive coping strategies, such as seeking social support (Pereira-Morales et al., 2018). Thus, OTE is demonstrably conducive not only to the promotion of art and creativity but also to health-related quality of life.

OTE has been reported to be a heritable personality trait. A meta-analysis of 100,000 pairs of twins and families showed that 41% of participants had higher levels of OTE than in other dimensions of personality (Vukasović and Bratko, 2015). Candidate gene studies have shown that OTE is associated with genes such as 5-hydroxy tryptamine transporter (5-HTT), the dopamine D4 receptor gene (DRD4) and the catechol-O-methyltransferase gene (COMT) (Kalbitzer et al., 2009; DeYoung et al., 2011). With the development of gene sequencing and analysis methods, genome-wide association studies (GWASs) and genome-wide polygenic scores (GPSs) have been used to explore the relationship between genes and phenotypes (Dudbridge, 2013). GWASs are based on a larger sample size, and, as the sample size increases, GWASs can detect more risk-related loci (International Schizophrenia Consortium, 2009). The GWAS for OTE conducted by the Genetics of Personality Consortium (GPC) identified 2 genetic loci achieving genome-wide statistical significance (De Moor et al., 2012). However, the effect size of phenotypic variation explained by SNPs obtained by GWAS is very small (Visscher et al., 2012). The locus can only explain 0.22% of the variation for OTE, with odds ratios generally between 0.92 and 1 (De Moor et al., 2012). GPSs have allowed us to use large GWAS power in small-sample applications (Dima and Breen, 2015). GPSs can be used to estimate the cumulative effects of SNPs associated with certain phenotypes in each individual, thereby exploring the genetic basis that may affect the individual phenotype (Plomin and von Stumm, 2018). GPSs of OTE have been reported to be correlated with symptoms of diseases (Amare et al., 2018) and cognitive traits (Abdellaoui et al., 2018). For example, researchers using a polygenic score of personality traits (including OTE) in depressive patients found a genetic association between the five personality traits and the outcomes of serotonin reuptake inhibitors (SSRIs) (Amare et al., 2018). GPSs of OTE can provide greater effects and avoid the loss of genetic effects.

There is a large body of research demonstrating that the brain structure and functions of psychological and personality factors. In structural magnetic resonance imaging (sMRI) studies, OTE has been reported to be related to the prefrontal cortex (PFC) (DeYoung et al., 2005), the gray matter volume of the inferior parietal lobule, the middle temporal gyrus and the lingual gyrus (DeYoung et al., 2010). Kapogiannis further revealed a negative correlation between OTE and the bilateral fusiform gyrus, right medial prefrontal cortex (OFC) and left insular lobe (Kapogiannis et al., 2013). In functional magnetic resonance imaging (fMRI) studies, most studies examine the resting-state functional connectivity. Beaty et al. (2016) found that OTE was significantly correlated with the functional connectivity of default network. Beaty and colleagues further explore the dynamic functional connectivity of OTE, and they found a significant association between OTE and the dynamic functional connectivity of default network and the cognitive control network (Beaty et al., 2018).

Above all, OTE research has always been a hot topic in gene and neuroimaging studies. Previous studies used candidate gene approaches to explore the genetic mechanism of OTE. However, OTE has been shown to be polygenic, and GPSs can provide greater genetic effects than other genetic analysis methods (Middeldorp et al., 2011). Moreover, numerous studies focused on behavioral genetics have directly explored the relationship between OTE and genes. As far as we know, however, previous studies have never examined the potential effects of OTE on this cumulative effect at the level of brain mechanisms. This model has been used primarily to examine the personality traits of normal people. We hypothesize that the neural phenotypes are related to brain structure and functions in some specific regions, such as the PFC, OFC, fusiform gyrus (Jauk et al., 2015), and in default network (Beaty et al., 2016); additionally, these regions had been reported previously in studies on OTE behavior research networks (Beaty et al., 2016). In the present study, we combined GPSs, resting-state fMRI and sMRI to determine the neural phenotypes influenced by OTE genetic variants.

## Materials and Methods

### Participants

In the present study, 586 college students were recruited from Southwest University in Chongqing (mean ± *SD* = 19.5 ± 1.5), including 180 males and 406 females. All participants were right-handed and healthy, with no history of neurological or psychological disorders and provided written informed consent. All participants were required to complete brain imaging data and genotyping data acquisition. This study was approved by the Southwest University Brain Imaging Center Institutional Review Board.

### Image Acquisition

All structural and functional MRI scans were performed on a 3T Trio scanner (Siemens Medical Systems, Erlangen, Germany) at the Brain Imaging Center, Southwest University.

The resting-state fMRI images were acquired using a gradient echo-planar imaging sequence: repetition time (TR)/echo time (TE) = 2,000 ms/30 ms, flip angle = 90°, slices = 32, thickness = 3 mm, field of view (FOV) = 240 × 240 mm^2^, resolution matrix = 64 × 64, voxel size = 3.4 × 3.4 × 3 mm^3^. High-resolution T1 structure images were acquired using a magnetization-prepared rapid gradient echo (MPRAGE) sequences: TR/TE = 1,900 ms/2.52 ms, inversion time = 900 ms, flip angle = 9°, slices = 176, thickness = l mm, FOV = 256 × 256 mm^2^, voxel size = 1 × l × l mm^3^.

### Image Preprocessing

The DPARSF software^1^ and the REST toolkit (Song et al., 2011) were based on MATLAB (Math Works, Natick, MA, United States), and SPM12 was used to preprocess the resting-state data. First, the first 10 volumes were discarded to allow the signal to reach equilibrium. Then, the remaining images were preprocessed by slice-time correction; head motion correction, removing the data of subjects whose head moved more than 2 mm on the X, Y, and Z axis or whose rotation angle exceeded 2°; spatial standardization and image registration to the Montreal Neurological Institute (MNI) standard template; resampling into 3 × 3 × 3 mm^3^; using a 6 mm FWHM Gaussian kernel for spatial smoothing to improve the signal-to-noise ratio of the image; removing linear drift; and using 0.01–0.08 Hz bandpass filtering to extract the low-frequency oscillation signalpart, eliminating physiological noise.

### Whole-Brain Functional Network Construction

We used ROIs based on the templates from Dosenbach to construct whole-brain network (Dosenbach et al., 2007). 160-region network definition has been shown to perform better than the 90-parcel AAL atlas and voxel-based graph in representing some aspects of the functional organization of the brain with fMRI data (Dosenbach et al., 2010). The time series of 160 regions were extracted by averaging the time series of each ROI, and Pearson correlation between each pair of ROIs was calculated, generating 12,720 FC edges.

### Cortical Surface Reconstruction and Cortical Thickness Measurement

Cortical surface reconstruction and cortical thickness measurements were obtained using the FreeSurfer toolkit^2^. This set of methods was originally proposed by Dale in the 1990s (Dale et al., 1999; Fischl et al., 1999a), and improvements over the years finally resulted in an automated process (Fischl et al., 1999b, 2001, 2002; Ségonne et al., 2004; Fischl, 2012). First, a spatial adaptive non-local mean filter was used to denoise structural images (T1 volume) and start skull stripping. Next, cerebrospinal fluid, white matter and gray matter are automatically segmented, and the cortical surface is reconstructed to produce a smooth representation of the GM-WM interface (white surface) and the GM-CSF (cortical surface). After normalized estimation of triangular mesh space, the mesh is transformed over the GM-WM boundary. The surface patch curvature is calculated; then, the surface is modified to ensure that the curvature of the local surface is at the most appropriate scale. Then, the first 5 EPI volumes of each participant are discarded, and the slice timing is corrected. To map the brains of all subjects into a common space, the reconstructed surfaces are recorded onto an average cortical surface map using a non-linear program that optimizes the cross-boundary alignment of sulcal and gyral features (Fischer and Getis, 1999).

### Genotyping

Approximately 2 μg genome DNA (gDNA) was extracted from 250 μl whole blood using an Axypre Blood Genomic DNA Kit (Corning Life Sciences, cat. no. 11313KC3). The concentration of all gDNA was quantified with the Qubit2.0 Fluorometer (Life Technologies, cat. no. Q32866) and the Qubit dsDNA HS Assay Kit (Life Technologies, cat. no. Q32854). gDNA samples were genotyped on the Illumina HumanExome v1.1 BeadChip (Illumina, San Diego, CA, United States) according to the manufacturer’s specifications. The genotyping module of Genomestudio v3.0 (Illumina, San Diego, CA, United States) was used to determine the genotypes based on the fluorescent signal with standard cluster algorithm.

### Quality Control and Imputation

Quality control of the genomic data was performed using PLINK v1.9^3^ (Purcell, 2009; Chang et al., 2015). First, individuals with discordant sex information, outlying heterozygosity rate (± 3 SD from the mean) and SNPs call rate < 0.98 were removed. Duplicated and high-relatedness (pi-hat > 0.185) individuals were also excluded. Then, SNPs were filtered out with the following criteria: missingness > 0.02, Hardy-Weinberg equilibrium *p* < 10^–4^, and minor allele frequency (MAF) < 0.05. To control for population stratification, a principal component analysis (PCA) was performed using EIGENSTRAT version 6.1.3 and the HapMap phase 3 reference data set (Price et al., 2006). After 10 principal components (PCs) were obtained, individuals with PC1 or PC2 ± 6 SD from the mean were removed. After quality control, the SNP data was imputed from the EAS population of the 1,000 Genomes Phase3 reference dataset using the Michigan Imputation Server^4^ (Das et al., 2016). Phasing and imputation 1,000 were conducted using Eagle v2.3 and Minimac4 (Loh et al., 2016). Imputed SNPs were excluded with info score (*R*^2^ < 0.6) and MAF < 0.01.

### OTE-Related Polygenic Analysis

GPSs of OTE were calculated using the “score” command in the PLINK software with reference to Purcell (2009). The reference GWAS statistics were derived from a Big Five personality GWAS meta-analysis published by the Genetics of Personality Consortium (De Moor et al., 2012)^5^. A total of 2.4 M single-nucleotide polymorphisms was found in 10 samples (17,375 adults) and five replicated samples (3,294 adults). From this result, we obtained the effect size of SNP risk alleles (obtained by logarithmic transformation of Odds Ratio value) and the correlation significance (*p*-value). Then, representative SNPs were selected from each linkage disequilibrium (LD) block, which made the loci included in the calculation of GPSs relatively independent. Finally, different GWAS association significance (*p* = 0.0001, 0.001, 0.01, 0.05, 0.25, 0.5, 1) was selected, and the risk effects of the corresponding reference sites were multiplied by the number of risk alleles (0, 1, or 2) carried by the subjects to obtain the GPSs under the seven threshold values. Previous studies have shown that a polygenic risk score (PRS) with a threshold of 0.05 generated the highest risk score prediction accuracy (Ripke et al., 2014). Another study also found that PRS6 (*p* = 0.05) constructed from schizophrenia-related alleles produced the highest risk score with prediction accuracy for hippocampal activity (Chen et al., 2018). Therefore, the GPSs with a threshold of 0.05 were selected for the main results. Other threshold results are shown in Supplementary Materials.

### Statistical Analysis

#### Association Between Cortical Thickness and GPSs of OTE

For each hemisphere, a multivariate general linear model (GLM) was used to test the correlation between each subject’s surface-based morphometry measures at each vertex and OTE GPSs. Age and gender were treated as the independent covariates of the GLM. A Monte Carlo simulation was used to control multiple comparisons (vertex-wise threshold of *p* < 0.001, cluster-wise threshold of *p* < 0.05) (Ledberg et al., 1998). Next, the cortical thickness values of brain regions significantly correlated with GPSs of OTE were extracted. Then, the cortical thickness and GPSs were then Z-transformed. Finally, correlation analysis was used to calculate the relationship between cortical thickness and GPSs.

#### Association Between Functional Connectivity and GPSs of OTE

Firstly, the Pearson correlation was calculated between each of the 12,720 (in the160 nodes network) edges and the GPSs of OTE after been transformed fisher Z across all subjects, and age and gender were treated as the independent covariates of the GLM. Afterward, the false discovery rate (FDR) (*p* < 0.05) in GRETNA software was used to control multiple comparisons.

## Results

### Relationship Between GPSs and Cortical Thickness

**FIGURE 1:**
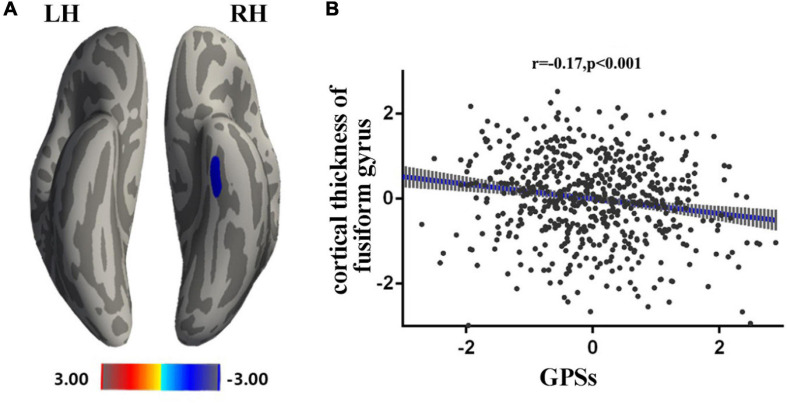
Associations between GPSs and cortical thickness. Statistical parametric maps from multiple linear regression analysis, after controlling for age and sex, demonstrated the correlation of GPSs and cortical thickness of the left fusiform [smooth = 10, Size (mm^2^) = 281.81, *X* = –37, *Y* = –17, *Z* = –25.30, NVtxs = 579]. The red bar denotes increased correlation between the cortical thickness of the left fusiform and GPSs after controlling for age and sex. The blue bar denotes decreased correlation between the cortical thickness of the left fusiform and GPSs after controlling for age and sex **(A)**. Scatter plots of the correlations between the significant regions and the GPSs **(B)**.

Multivariate regression was used to establish a statistical model in 586 subjects. Sex and age were used as covariates to calculate the correlation between cortical thickness and GPSs (*p* = 0.05 for example, other thresholds in Supplementary Material). After performing Monte Carlo simulation (vertex-wise threshold of *p* < 0.001, cluster-wise threshold of *p* < 0.05), a cluster survived—fusiform [size (mm^2^) = 281.81, *X* = −37, *Y* = −17, *Z* = −25.30, NVtxs = 579] (Figure 1).

### Relationship Between GPSs and Functional Connectivity

**FIGURE 2:**
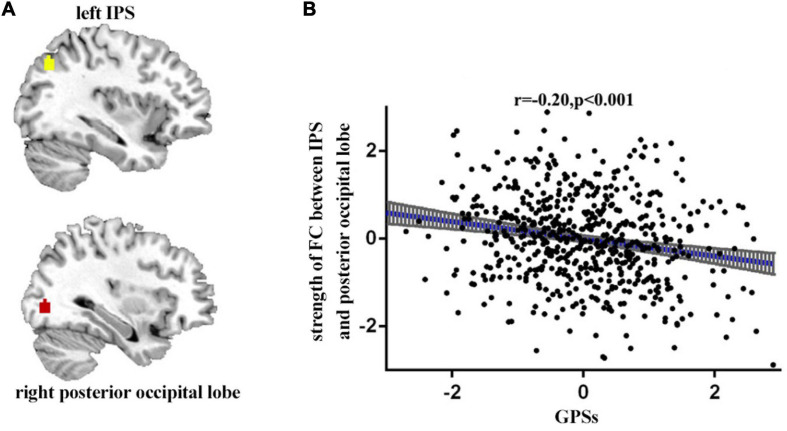
The functional connectivity between the left IPS (*x* = –36, *y* = –69, *z* = 40) and the right posterior occipital lobe (*x* = 33, *y* = –81, *z* = v2). Participants with high GPSs showed significantly lower RSFC in these regions **(A)**. Scatter plots of the correlations between GPSs and the functional connectivity **(B)**.

We then examined the associations between GPSs and the strength of functional connectivity. Age and gender were treated as the covariates, the Pearson correlation analysis was performed between GPSs and functional connectivity. The results showed a negative association between GPSs of OTE and the functional connectivity between the left IPS (*x* = −36, *y* = −69, *z* = 40) and the right posterior occipital lobe (*x* = 33, *y* = −81, *z* = −2) (*p* < 0.001, *r* = −0.20) (Figure 2).

## Discussion

The present study examined the relationship between the GPSs of OTE and cortical thickness as well as for intrinsic functional connectivity. The higher GPSs were significantly associated with a thinner fusiform gyrus and a weaker functional connectivity between IPS and the right posterior occipital lobe. Studies of behavioral genetics have indicated that a single gene directly acts on OTE, such as DRD4 and 5-HTT (Kalbitzer et al., 2009; DeYoung et al., 2011). The present results suggested that OTE, a complex personality trait, was influenced by multiple genes and mediated by the structure and function of the brain. The present study firstly combined neuroimaging data and gene data to identify two neural phenotypes in a region shown in previous studies to be related to OTE. Stimulating these phenotypes may influence the relationship between genes and OTE, thereby achieving the goal of improving OTE. The current findings could deepen our understanding of the internal mechanism of the formation of the OTE personality trait and can also help with the intervention in and cultivation of an open personality.

In the present study, a negative correlation between the GPSs of OTE and the cortical thickness of the fusiform gyrus were observed. The fusiform gyrus, located at the middle and bottom of the visual syncortex, is generally considered to be involved in face recognition and secondary classification of objects (McCarthy et al., 1997; Joseph and Gathers, 2002; Ramírez et al., 2014). Kapogiannis et al. (2013) found a negative correlation between OTE and the bilateral fusiform gyrus, right medial prefrontal cortex (OFC) and left insular lobe. Vivid visual stimuli have been associated with the activation of fusiform and temporal lobe (Wheeler et al., 2000). Moreover, individuals with high OTE tend to seek novel experiences and feelings through their senses (Aluja et al., 2003). The thinning fusiform gyrus may not be a biomarker of cognitive impairment but, rather, of the cognitive disinhibition of flexible cognition (Vartanian et al., 2018). Therefore, the present findings may suggest that people with higher GPSs of OTE have thicker spindle gyrus pruning, which allows for faster information flows, higher cognitive flexibility, and enhanced sensitivity to new stimuli. Furthermore, an important characteristic of the OTE population has been noted as high creativity (McCrae, 1987). Diminishing cortical thickness in areas including fusiform gyrus, left frontal lobe, lingual, and cuneus is associated with increased creativity (Jung et al., 2010). The mechanism of cortical thinning has been thought to involve more centralized functional activation in skill acquisition to improve information efficiency (Durston and Casey, 2006). In brief, the present study suggests that reduced cortical thickness in the fusiform gyrus may associated with fewer information barriers and increased cognitive flexibility, and the thinner fusiform gyrus is a gene-mediated intermediate phenotype for high OTE.

Importantly, the present study has shown that GPSs of OTE were negatively associated with the functional connectivity between the IPS and the right posterior occipital lobe. IPS is related mainly to visual perception, cognitive flexibility, guidance of attention, inhibitory control and working memory (Duhamel et al., 1998; Rottschy et al., 2012; Cieslik et al., 2013; Müller et al., 2015; Christopoulos et al., 2018). Higher OTE is associated with higher cognitive flexibility (DeYoung et al., 2005). Previous studies have shown that OTE is related to IPS. For example, Wright and colleagues reported a negative correlation between OTE/intelligence and inferior parietal cortical thickness in healthy elderly participants (Wainwright et al., 2008). Vartanian et al. (2018) also found that left hemispheric cortical thickness was negatively correlated with OTE, which was shown to be true for the left MTG, superior temporal gyrus, inferior parietal lobule and middle frontal gyrus (). Therefore, the present findings suggest that IPS influences GPSs of OTE through cognitive flexibility. Furthermore, IPS is located in the default network (Dosenbach et al., 2010). Previous study showed that the default network (DN) was related to OTE with more effective information processing (Patel et al., 2012). DN activity has been associated with spontaneous cognitive processes, such as autobiographical memory retrieval, future imagery, mental reasoning theory, moral decision-making judgment, creative cognition, and meditation, which are consistent with OTE (Beaty et al., 2016). Variations in the default network may be crucial to understanding individual differences in OTE (DeYoung and Allen, 2019). The occipital lobe is responsible for processing language, motion sense, abstract concepts and visual information (Kato et al., 1993; Bedny et al., 2011). An event-related potential (ERP) study showed greater theta event-related synchronization in the parietal and occipital regions, particularly on the right side on a sample of high OTE (Aftanas et al., 2001). Zhiyan and Singer (1997) demonstrated that people with high OTE scores were more likely to enter into mind wandering in visual tasks and demonstrate less attention. Individuals with high GPSs of OTE were more likely to diverge from outside visual stimuli through visual areas such as the occipital lobe to generate novel ideas. Furthermore, the parietal and occipital regions tend to be disconnected from the right temporal region when highly open groups engage in fantasies or generating new ideas (Camfield, 2008). It has also been demonstrated that the lower functional connectivity between the occipital and parietal is associated with, the lower exogenous visual top-down attention effect (Tsubomi et al., 2009). Previous findings suggest that the connections between the IPS and the occipital posterior lobe is partly regulated by PGSs of OTE. The weaker connections between IPS and posterior occipital lobe may be associated with lower attention and better access to mind wandering and generation of new ideas.

Finally, the present study has several limitations. First, the GWAS data used in this study to construct the polygenic score was obtained from the GPC, which is a database of European samples. However, the genetic data involved in the current study are from Chinese sample, and the calculation of polygenic score may be biased by race and culture (Liu et al., 2017). However, to our knowledge, there is no large gene database of Asian personality traits. Future research could establish Chinese genetic database to improve the statistical power of polygenic scores. Furthermore, the participants involved in the present study have high homogeneity. The age of participants is concentrated in the 18–20 range. Therefore, the funding of the present study is difficult to generalize to other age groups. Future research could examine individual differences in genetic and brain mechanisms on a wide age range sample.

## Conclusion

In summary, our study suggested that the GPSs of OTE were negatively correlated with the cortical thickness of the fusiform gyrus and with the functional connectivity between the IPS and the posterior occipital lobe. These findings provide new insights that improve our understanding of the relationship between the intermediate phenotype of the brain biomarkers and genetic architecture of OTE.

## Data Availability Statement

The datasets presented in this article are not readily available because: This article contains genetic data and is not transferable. Requests to access the datasets should be directed to qiuj318@swu.edu.cn">qiuj318@swu.edu.cn.

## Ethics Statement

The studies involving human participants were reviewed and approved by the Southwest University Brain Imaging Center Institutional Review Board. The patients/participants provided their written informed consent to participate in this study.

## Author Contributions

ZR, CL, and JM analyzed the data and wrote the manuscript. ZR revised the manuscript and responsed to reviewers. JQ proposed the idea of the study and drafted the outline of the manuscript. QL and LS offered technical assistance in analyzing data. XW and LS proposed the idea of data analysis. All authors contributed to data acquisition.

## Conflict of Interest

The authors declare that the research was conducted in the absence of any commercial or financial relationships that could be construed as a potential conflict of interest.

## Footnotes

1 http://resting-fmri.sourceforge.net/

2 http://surfer.nmr.mgh.harvard.edu/

3 https://www.cog-genomics.org/plink/1.9/

4 https://imputationserver.sph.umich.edu/index.html

5 http://www.tweelingenregister.org/GPC/
