# Supplementary material for: Effects of the Openness to Experience Polygenic Score on Cortical Thickness and Functional Connectivity
Source: Front Neurosci. 2021 Jan 11;14:607912. doi: 10.3389/fnins.2020.607912 (PMC7829912; doi:10.3389/fnins.2020.607912)
Supplement: Supplementary file 1 [file Table_1.DOCX]

**Supplementary Table A. The result for association analysis of openness GPSs and cortical thickness (smooth=10,** **vertex-wise forming threshold of p<0.001, cluster-wise forming threshold of p<0.05)**

| threshold | Size(mm^2) | X | Y | Z | NVtxs | Annot |
| --- | --- | --- | --- | --- | --- | --- |
| 0.05 | 281.81 | -37 | -17 | -25.30 | 579 | fusiform |
| 0.25 | 435.19 | -38.60 | -16.50 | -23.90 | 817 | fusiform |
| 0.5 | 423.03 | -39.30 | -18.90 | -22.70 | 800 | fusiform |
| 1 | 479.32 | -39.30 | -18.90 | -22.70 | 904 | fusiform |

**Supplementary Table B. The result for association analysis of openness GPSs and cortical thickness (smooth=15,** **vertex-wise forming threshold of p<0.001, cluster-wise forming threshold of p<0.05)**

| threshold | Size(mm^2) | X | Y | Z | NVtxs | Annot |
| --- | --- | --- | --- | --- | --- | --- |
| 0.05 | 539.33 | -37.60 | -14.80 | -25.60 | 1000 | fusiform |
| 0.25 | 648.84 | -38.50 | -14.60 | -25 | 1209 | fusiform |
| 0.5 | 665.99 | -39.20 | -15.90 | -24 | 1242 | fusiform |
| 1 | 724.95 | -39.30 | -16.80 | -23.50 | 1347 | fusiform |

**Supplementary Table C. The result for association analysis of openness GPSs and cortical thickness (threshold p=0.0001, uncorrected)**

| ClusterNo | Size(mm^2) | X | Y | Z | NVtxs | Annot |
| --- | --- | --- | --- | --- | --- | --- |
| 1 | 398.6 | -54.50 | -26.80 | 32.80 | 951 | supramarginal |
| 2 | 1173.25 | -4.20 | -16.10 | 30.50 | 2982 | posteriorcingulate |
| 3 | 342.2 | -40.90 | -69.00 | 25.90 | 597 | inferiorparietal |
| 4 | 286.41 | -34.70 | 51.20 | -0.90 | 401 | rostralmiddlefrontal |
| 5 | 246.05 | -35.40 | -5.60 | 41.70 | 470 | precentral |
| 6 | 157.21 | -41.90 | -69.70 | -2.80 | 266 | lateraloccipital |
| 7 | 437.6 | -37.60 | 8.20 | -26.60 | 884 | superiortemporal |
| 8 | 201.02 | -8 | -88.20 | 18.10 | 211 | cuneus |
| 9 | 189.64 | -7.50 | 58.20 | -6.70 | 268 | medialorbitofrontal |
| 10 | 272.46 | -20.40 | -93.70 | 7.70 | 321 | lateraloccipital |
| 11 | 151.48 | -40.70 | -63.10 | 42.30 | 338 | inferiorparietal |
| 12 | 202.03 | -15.20 | -34 | 69.80 | 491 | postcentral |

**Supplementary Table D. The result for association analysis of openness GPSs and cortical thickness (threshold p=0.001, uncorrected)**

| ClusterNo | Size(mm^2) | X | Y | Z | NVtxs | Annot |
| --- | --- | --- | --- | --- | --- | --- |
| 1 | 417.43 | -55.10 | -26.40 | 32.00 | 979 | supramarginal |
| 2 | 473.61 | -22.10 | -53.50 | 7.10 | 1126 | lingual |
| 3 | 1250.72 | -7.50 | 58.20 | -6.70 | 1773 | medialorbitofrontal |
| 4 | 334.67 | -35.20 | -5.90 | 43.00 | 680 | precentral |
| 5 | 287.17 | -4 | -23 | 31.10 | 838 | posteriorcingulate |
| 6 | 413.01 | -39.90 | -62.40 | 35 | 817 | inferiorparietal |
| 7 | 152.66 | -23.30 | 22.10 | 46 | 209 | superiorfrontal |
| 8 | 185.51 | -22.10 | -90.40 | 7.50 | 238 | lateraloccipital |

**Supplementary Table E. The result for association analysis of openness GPSs and cortical thickness (threshold p=0.01, uncorrected)**

| ClusterNo | Size(mm^2) | X | Y | Z | NVtxs | Annot |
| --- | --- | --- | --- | --- | --- | --- |
| 1 | 317.99 | -23.20 | 21.20 | 47.80 | 459 | superiorfrontal |
| 2 | 1252.43 | -7.90 | 56.40 | -5.9 | 1787 | medialorbitofrontal |
| 3 | 540.51 | -53.20 | -21.70 | -23.9 | 836 | inferiortemporal |
| 4 | 248.92 | -36.80 | -5.60 | 43.40 | 475 | precentral |
| 5 | 194.67 | -4.70 | -7.30 | 28.10 | 570 | posteriorcingulate |
| 6 | 175.32 | -22.40 | 53.60 | 13.50 | 221 | rostralmiddlefrontal |
| 7 | 348.90 | -47.90 | -17 | -0.80 | 707 | superiortemporal |
| 8 | 209.97 | -55.30 | -16.60 | 43.60 | 421 | postcentral |
